# A new mixed group 5 metal selenide, Nb_1.41_V_0.59_Se_9_

**DOI:** 10.1107/S1600536811033319

**Published:** 2011-08-27

**Authors:** Eunsil Lee, Hoseop Yun

**Affiliations:** a Division of Energy Systems Research and Department of Chemistry, Ajou University, Suwon 443-749, Republic of Korea

## Abstract

The new mixed-metallic phase, niobium vanadium nona­selenide, (Nb_2-*x*_V_*x*_)Se_9_ (0.18≤ *x* ≤ 0.59) is isostructural with monoclinic V_2_Se_9_. The structure is composed of chains of bicapped trigonal–prismatic [*M*Se_8_] units. The metal (*M*) site is occupied by statistically disordered Nb [0.706 (5)] and V [0.294 (5)] atoms. Two trigonal prisms are linked by sharing a recta­ngular face composed of two Se_2_^2−^ pairs. Through three edging and capping Se atoms, the chains are extended along [101]. The chain shows alternating short [2.8847 (7) Å] and long [3.7159 (7) Å] *M*—*M* distances. The structure shows a wide range of Se—Se inter­actions. In addition to the Se_2_^2−^ pairs of the recta­ngular face, an inter­mediate Se⋯Se separation [2.6584 (5) Å] is found. The amount of each metal can vary, [(Nb_2-*x*_V_*x*_)Se_9_, 0.18 ≤ *x* ≤m 0.59] and they seem to form a random substitutional solid solution. The *M*—*M* distances increase gradually by increasing the amount of Nb atoms. The classical charge-balance of the compound can be described as [*M* ^4+^]_2_[Se_2_^2−^]_2_[Se_5_^4−^].

## Related literature

For related group 5 metal chalcogenide triclinic Nb_2_Se_9_ structures, see: Meerschaut *et al.* (1979 ▶); Sunshine & Ibers (1987 ▶). For the synthesis and structures of related group 5 metal monoclinic V_2_Se_9_ chalcogenides, see: Furuseth & Klewe (1984 ▶).

## Experimental

### 

#### Crystal data

Nb_1.41_V_0.59_Se_9_
*M* *_r_* = 871.69
Monoclinic, 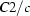

*a* = 10.8039 (5) Å
*b* = 12.6209 (7) Å
*c* = 8.1704 (3) Å
β = 94.6473 (15)°
*V* = 1110.41 (9) Å^3^
*Z* = 4
Mo *K*α radiation
μ = 31.39 mm^−1^
*T* = 290 K
0.36 × 0.02 × 0.02 mm

#### Data collection

Rigaku R-AXIS RAPID diffractometer
Absorption correction: multi-scan (*NUMABS*; Higashi, 2000 ▶) *T*_min_ = 0.415, *T*_max_ = 1.000
5325 measured reflections
1281 independent reflections
1141 reflections with *I* > 2σ(*I*)
*R*_int_ = 0.038

#### Refinement

*R*[*F* ^2^ > 2σ(*F* ^2^)] = 0.022
*wR*(*F* ^2^) = 0.051
*S* = 1.12
1281 reflections
52 parameters
Δρ_max_ = 1.07 e Å^−3^
Δρ_min_ = −0.72 e Å^−3^

### 

Data collection: *RAPID-AUTO* (Rigaku, 2006 ▶); cell refinement: *RAPID-AUTO*; data reduction: *RAPID-AUTO*; program(s) used to solve structure: *SHELXS97* (Sheldrick, 2008 ▶); program(s) used to refine structure: *SHELXL97* (Sheldrick, 2008 ▶); molecular graphics: locally modified version of *ORTEP* (Johnson, 1965 ▶); software used to prepare material for publication: *WinGX* (Farrugia, 1999 ▶).

## Supplementary Material

Crystal structure: contains datablock(s) global, I. DOI: 10.1107/S1600536811033319/jj2093sup1.cif

Structure factors: contains datablock(s) I. DOI: 10.1107/S1600536811033319/jj2093Isup2.hkl

Additional supplementary materials:  crystallographic information; 3D view; checkCIF report

## Figures and Tables

**Fig. 1.**
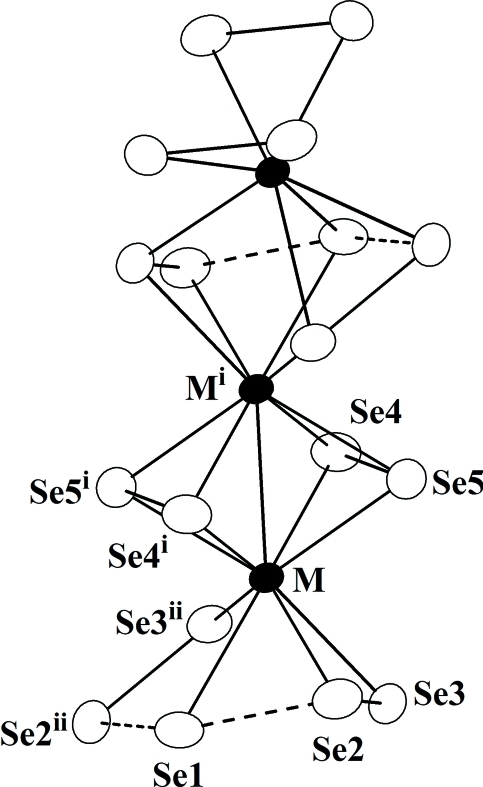
A view of the chain fragment of (Nb1.41V0.59)Se9. Displacement ellipsoids are drawn at the 80% probability level. [Symmetry code: (i) -x + 1/2, -y + 1/2, -z + 1; (ii) -x, y, -z + 1/2]

**Fig. 2.**
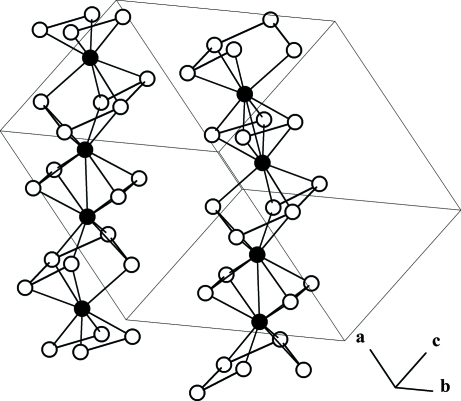
The structure of the one-dimensional (Nb1.41V0.59)Se9. Atoms are as marked in Fig. 1.

**Fig. 3.**
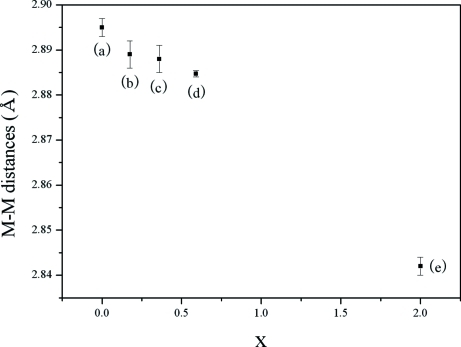
Plots of M—M distances (Å) versus x in (Nb2 -xVx)Se9. [(a) x = 0 (Sunshine & Ibers, 1987); (b) x = 0.18; (c) x = 0.36; (d) x = 0.59; (e) x = 2 (Furuseth & Klewe, 1984)]

## supplementary crystallographic information

### Comment

Group 5 metal chalcogenides usually have low-dimensional structures. In particular, both triclinic Nb_2_Se_9_ (Meerschaut *et al.*, 1979; Sunshine & Ibers, 1987) and monoclinic V_2_Se_9_ (Furuseth & Klewe, 1984) have been reported to have one-dimensional chain structures. These compounds share the same one-dimensional chain structure. However they show different packing of the chains and the twofold rotational symmetry is not observed in Nb_2_Se_9_. During our search for new group 5 metal chalcogenides, we have found a new mixed-metallic phase, (Nb_2 -_*_x_*V*_x_*)Se_9_ (0.18≤*x*≤0.59) and here we report the synthesis and crystal structure of (Nb_1.41_V_0.59_)Se_9_.

The title compound is isostructural with monoclinic V_2_Se_9_ (Furuseth & Klewe, 1984). The structure is composed of one-dimensional chains made of the bicapped trigonal prismatic [*M*_2_Se_10_] unit. The metal (*M*) site is occupied by statistically disordered Nb (0.706 (5) %) and V (0.294 (5) %) atoms. The Se atoms are found as Se_2_ or Se_5_ units. Each Nb atom is surrounded by two Se_2_ and one Se_5_ units. Two trigonal prisms are linked by sharing a rectangular face composed of two Se_2_^2-^ pairs (Fig. 1). Through three edging and capping Se atoms of the Se_5_ unit, the chains are extended along [101] (Fig. 2). The shortest interchain Se—Se distance is 3.5479 (8) Å and thus there is no strong bonding interaction among the chains. The chain shows alternating short (2.8847 (7) Å) and long (3.7159 (7) Å) M—*M* distances. The short M—*M* distance is in-between those found in V_2_Se_9_ (2.842 (2) Å, Furuseth & Klewe, 1984) and Nb_2_Se_9_ (2.895 (2) Å, Sunshine & Ibers, 1987). The structure shows a wide range of Se—Se interactions. In the prism, two Se4—Se5 pairs (2.3140 (6) Å) forming a rectangular face exhibit the regular Se—Se bonds. In addition, the intermediate Se1···Se2 separation (2.6584 (5) Å) is found along with the short Se2—Se3 distance (2.3603 (6) Å) in the Se_5_ unit.

The structural investigations of the three different crystals from the same reaction tube showed that the stoichiometries of each metal can vary, [(Nb_2-_*_x_*V*_x_*)Se_9,_ 0.18≤*x*≤0.59] and they seem to form a random substitutional solid solution. The intermetallic distances are affected by the contribution of each constituent metal. The M—*M* distances increase gradually by increasing the amount of Nb atoms (Fig.3). The classical charge balance of the compound can be described as [*M*^4+^]_2_[Se_2_^2-^]_2_[Se_5_^4-^].

### Experimental

The title compound, (Nb_1.41_V_0.59_)Se_9_ was prepared by the reaction of elements Nb, V, and Se at 873 K. A combination of the pure elements, Nb powder (CERAC 99.999%), V powder (Aldrich 99.5%), and Se powder (Aldrich 99.999%) were mixed in a fused silica tube in molar ratio of Nb: V: Se = 2: 1: 16. The tube was evacuated to 0.133 Pa, sealed, and heated gradually (20 K/h) to 873 K, where it was kept for 72 h. The tube was cooled to room temperature at the rate 3 K/h. The products were obtained as shiny black needle-shaped crystals. The crystals are stable in air and water. XRF analysis indicated the presence of Nb, V, and Se. Both X-ray diffraction studies and quantitative XRF analysis indicated that stoichiometries of each metal vary considerably for crystals even from the same reaction tube. The average Nb: V ratio for many crystals is 78: 22.

### Refinement

The disordered nature of the metals in the title compound was checked by refining the anisotropic displacement parameters (ADPs). When the structure was refined assuming Nb_2_Se_9_ and V_2_Se_9_, the displacement parameters of the metal sites were very large and small, respectively. In both cases the reliability indices were high (wR2 > 0.098). With the mixed-metal model, the ADPs of the metal atoms are comparable with those of the other atoms and the residuals were reduced significantly (wR2 = 0.051). The Se atoms were refined anisotropically.

### Crystal data

**Table:**

Nb_1.41_V_0.59_Se_9_	*F*(000) = 1508.8
*M**_r_* = 871.69	*D*_x_ = 5.215 Mg m^−^^3^
Monoclinic, *C*2/*c*	Mo *K*α radiation, λ = 0.71073 Å
Hall symbol: -C 2yc	Cell parameters from 4525 reflections
*a* = 10.8039 (5) Å	θ = 3.2–27.5°
*b* = 12.6209 (7) Å	µ = 31.39 mm^−^^1^
*c* = 8.1704 (3) Å	*T* = 290 K
β = 94.6473 (15)°	Needle, black
*V* = 1110.41 (9) Å^3^	0.36 × 0.02 × 0.02 mm
*Z* = 4	

### Data collection

**Table:**

Rigaku R-AXIS RAPID diffractometer	1281 independent reflections
Radiation source: fine-focus sealed tube	1141 reflections with *I* > 2σ(*I*)
graphite	*R*_int_ = 0.038
ω scans	θ_max_ = 27.5°, θ_min_ = 3.2°
Absorption correction: multi-scan (*NUMABS*; Higashi, 2000)	*h* = −11→14
*T*_min_ = 0.415, *T*_max_ = 1.000	*k* = −16→16
5325 measured reflections	*l* = −10→10

### Refinement

**Table:**

Refinement on *F*^2^	52 parameters
Least-squares matrix: full	0 restraints
*R*[*F*^2^ > 2σ(*F*^2^)] = 0.022	*w* = 1/[σ^2^(*F*_o_^2^) + (0.0198*P*)^2^ + 0.9634*P*] where *P* = (*F*_o_^2^ + 2*F*_c_^2^)/3
*wR*(*F*^2^) = 0.051	(Δ/σ)_max_ = 0.001
*S* = 1.12	Δρ_max_ = 1.07 e Å^−^^3^
1281 reflections	Δρ_min_ = −0.72 e Å^−^^3^

### Special details

**Table:**

Geometry. All s.u.'s (except the s.u. in the dihedral angle between two l.s. planes) are estimated using the full covariance matrix. The cell s.u.'s are taken into account individually in the estimation of s.u.'s in distances, angles and torsion angles; correlations between s.u.'s in cell parameters are only used when they are defined by crystal symmetry. An approximate (isotropic) treatment of cell s.u.'s is used for estimating s.u.'s involving l.s. planes.
Refinement. Refinement of *F*^2^ against ALL reflections. The weighted *R*-factor *wR* and goodness of fit *S* are based on *F*^2^, conventional *R*-factors *R* are based on *F*, with *F* set to zero for negative *F*^2^. The threshold expression of *F*^2^ > 2σ(*F*^2^) is used only for calculating *R*-factors(gt) *etc*. and is not relevant to the choice of reflections for refinement. *R*-factors based on *F*^2^ are statistically about twice as large as those based on *F*, and *R*- factors based on ALL data will be even larger.

### Fractional atomic coordinates and isotropic or equivalent isotropic displacement parameters (Å^2^)

**Table:**

	*x*	*y*	*z*	*U*_iso_*/*U*_eq_	Occ. (<1)
Nb1	0.13016 (4)	0.26771 (3)	0.41322 (4)	0.01363 (17)	0.706 (5)
V1	0.13016 (4)	0.26771 (3)	0.41322 (4)	0.01363 (17)	0.294 (5)
Se1	0	0.41770 (5)	0.25	0.02302 (15)	
Se2	−0.06049 (4)	0.35079 (4)	0.54157 (5)	0.02270 (13)	
Se3	−0.10370 (4)	0.19147 (4)	0.39677 (5)	0.01982 (12)	
Se4	0.23604 (4)	0.08172 (4)	0.44427 (5)	0.02230 (12)	
Se5	0.16278 (4)	0.15311 (4)	0.67875 (5)	0.02290 (12)	

### Atomic displacement parameters (Å^2^)

**Table:**

	*U*^11^	*U*^22^	*U*^33^	*U*^12^	*U*^13^	*U*^23^
Nb1	0.0106 (2)	0.0172 (3)	0.0130 (2)	0.00056 (16)	0.00004 (14)	0.00019 (15)
V1	0.0106 (2)	0.0172 (3)	0.0130 (2)	0.00056 (16)	0.00004 (14)	0.00019 (15)
Se1	0.0181 (3)	0.0191 (3)	0.0305 (3)	0	−0.0063 (2)	0
Se2	0.0191 (2)	0.0305 (3)	0.0185 (2)	0.00413 (18)	0.00163 (15)	−0.00470 (17)
Se3	0.0163 (2)	0.0254 (3)	0.0177 (2)	−0.00295 (18)	0.00143 (14)	0.00224 (16)
Se4	0.0207 (2)	0.0198 (2)	0.0256 (2)	0.00210 (18)	−0.00346 (16)	−0.00309 (17)
Se5	0.0180 (2)	0.0314 (3)	0.0191 (2)	−0.00145 (19)	0.00056 (14)	0.00614 (17)

### Geometric parameters (Å, °)

**Table:**

Nb1—Se4^i^	2.6046 (6)	Se1—Nb1^ii^	2.6524 (6)
Nb1—Se2	2.6061 (6)	Se1—Se2^ii^	2.6584 (5)
Nb1—Se5	2.6075 (6)	Se1—Se2	2.6584 (5)
Nb1—Se4	2.6146 (6)	Se2—Se3	2.3603 (6)
Nb1—Se5^i^	2.6153 (6)	Se3—V1^ii^	2.7028 (5)
Nb1—Se1	2.6524 (6)	Se3—Nb1^ii^	2.7028 (5)
Nb1—Se3	2.6968 (6)	Se4—Se5	2.3140 (6)
Nb1—Se3^ii^	2.7028 (5)	Se4—V1^i^	2.6046 (6)
Nb1—V1^i^	2.8847 (7)	Se4—Nb1^i^	2.6046 (6)
Nb1—Nb1^i^	2.8847 (7)	Se5—V1^i^	2.6153 (6)
Se1—V1^ii^	2.6524 (6)	Se5—Nb1^i^	2.6153 (6)
			
Se4^i^—Nb1—Se2	87.402 (19)	Se4^i^—Nb1—Nb1^i^	56.614 (16)
Se4^i^—Nb1—Se5	90.002 (18)	Se2—Nb1—Nb1^i^	124.88 (2)
Se2—Nb1—Se5	86.924 (18)	Se5—Nb1—Nb1^i^	56.603 (16)
Se4^i^—Nb1—Se4	112.894 (17)	Se4—Nb1—Nb1^i^	56.281 (17)
Se2—Nb1—Se4	132.53 (2)	Se5^i^—Nb1—Nb1^i^	56.345 (16)
Se5—Nb1—Se4	52.604 (16)	Se1—Nb1—Nb1^i^	140.90 (3)
Se4^i^—Nb1—Se5^i^	52.629 (16)	Se3—Nb1—Nb1^i^	140.29 (3)
Se2—Nb1—Se5^i^	133.17 (2)	Se3^ii^—Nb1—Nb1^i^	115.34 (2)
Se5—Nb1—Se5^i^	112.948 (17)	V1^i^—Nb1—Nb1^i^	0.00 (2)
Se4—Nb1—Se5^i^	89.611 (19)	V1^ii^—Se1—Nb1	88.93 (3)
Se4^i^—Nb1—Se1	87.508 (19)	Nb1^ii^—Se1—Nb1	88.93 (3)
Se2—Nb1—Se1	60.729 (13)	V1^ii^—Se1—Se2^ii^	58.774 (15)
Se5—Nb1—Se1	147.63 (2)	Nb1^ii^—Se1—Se2^ii^	58.774 (15)
Se4—Nb1—Se1	154.09 (2)	Nb1—Se1—Se2^ii^	93.73 (2)
Se5^i^—Nb1—Se1	90.793 (17)	V1^ii^—Se1—Se2	93.73 (2)
Se4^i^—Nb1—Se3	140.03 (2)	Nb1^ii^—Se1—Se2	93.73 (2)
Se2—Nb1—Se3	52.827 (16)	Nb1—Se1—Se2	58.774 (15)
Se5—Nb1—Se3	84.635 (18)	Se2^ii^—Se1—Se2	142.96 (3)
Se4—Nb1—Se3	94.89 (2)	Se3—Se2—Nb1	65.560 (17)
Se5^i^—Nb1—Se3	160.346 (19)	Se3—Se2—Se1	82.79 (2)
Se1—Nb1—Se3	76.889 (16)	Nb1—Se2—Se1	60.497 (15)
Se4^i^—Nb1—Se3^ii^	133.74 (2)	Se2—Se3—Nb1	61.613 (16)
Se2—Nb1—Se3^ii^	119.558 (18)	Se2—Se3—V1^ii^	99.67 (2)
Se5—Nb1—Se3^ii^	125.39 (2)	Nb1—Se3—V1^ii^	86.973 (17)
Se4—Nb1—Se3^ii^	77.498 (17)	Se2—Se3—Nb1^ii^	99.67 (2)
Se5^i^—Nb1—Se3^ii^	83.958 (17)	Nb1—Se3—Nb1^ii^	86.973 (17)
Se1—Nb1—Se3^ii^	76.786 (16)	Se5—Se4—V1^i^	63.925 (18)
Se3—Nb1—Se3^ii^	78.391 (18)	Se5—Se4—Nb1^i^	63.925 (18)
Se4^i^—Nb1—V1^i^	56.614 (16)	Se5—Se4—Nb1	63.540 (17)
Se2—Nb1—V1^i^	124.88 (2)	V1^i^—Se4—Nb1	67.105 (17)
Se5—Nb1—V1^i^	56.603 (16)	Nb1^i^—Se4—Nb1	67.105 (17)
Se4—Nb1—V1^i^	56.281 (17)	Se4—Se5—Nb1	63.856 (18)
Se5^i^—Nb1—V1^i^	56.345 (16)	Se4—Se5—V1^i^	63.447 (18)
Se1—Nb1—V1^i^	140.90 (3)	Nb1—Se5—V1^i^	67.052 (17)
Se3—Nb1—V1^i^	140.29 (3)	Se4—Se5—Nb1^i^	63.447 (18)
Se3^ii^—Nb1—V1^i^	115.34 (2)	Nb1—Se5—Nb1^i^	67.052 (17)
			
Se4^i^—Nb1—Se1—V1^ii^	176.568 (19)	Se2—Nb1—Se3—V1^ii^	102.699 (18)
Se2—Nb1—Se1—V1^ii^	−95.014 (17)	Se5—Nb1—Se3—V1^ii^	−167.096 (18)
Se5—Nb1—Se1—V1^ii^	−97.37 (4)	Se4—Nb1—Se3—V1^ii^	−115.431 (19)
Se4—Nb1—Se1—V1^ii^	33.29 (4)	Se5^i^—Nb1—Se3—V1^ii^	−12.76 (8)
Se5^i^—Nb1—Se1—V1^ii^	124.029 (18)	Se1—Nb1—Se3—V1^ii^	39.663 (15)
Se3—Nb1—Se1—V1^ii^	−40.511 (11)	Se3^ii^—Nb1—Se3—V1^ii^	−39.28 (2)
Se3^ii^—Nb1—Se1—V1^ii^	40.424 (11)	V1^i^—Nb1—Se3—V1^ii^	−155.17 (3)
V1^i^—Nb1—Se1—V1^ii^	154.52 (4)	Nb1^i^—Nb1—Se3—V1^ii^	−155.17 (3)
Nb1^i^—Nb1—Se1—V1^ii^	154.52 (4)	Se4^i^—Nb1—Se3—Nb1^ii^	109.33 (2)
Se4^i^—Nb1—Se1—Nb1^ii^	176.568 (19)	Se2—Nb1—Se3—Nb1^ii^	102.699 (18)
Se2—Nb1—Se1—Nb1^ii^	−95.014 (17)	Se5—Nb1—Se3—Nb1^ii^	−167.096 (18)
Se5—Nb1—Se1—Nb1^ii^	−97.37 (4)	Se4—Nb1—Se3—Nb1^ii^	−115.431 (19)
Se4—Nb1—Se1—Nb1^ii^	33.29 (4)	Se5^i^—Nb1—Se3—Nb1^ii^	−12.76 (8)
Se5^i^—Nb1—Se1—Nb1^ii^	124.029 (18)	Se1—Nb1—Se3—Nb1^ii^	39.663 (15)
Se3—Nb1—Se1—Nb1^ii^	−40.511 (11)	Se3^ii^—Nb1—Se3—Nb1^ii^	−39.28 (2)
Se3^ii^—Nb1—Se1—Nb1^ii^	40.424 (11)	V1^i^—Nb1—Se3—Nb1^ii^	−155.17 (3)
V1^i^—Nb1—Se1—Nb1^ii^	154.52 (4)	Nb1^i^—Nb1—Se3—Nb1^ii^	−155.17 (3)
Nb1^i^—Nb1—Se1—Nb1^ii^	154.52 (4)	Se4^i^—Nb1—Se4—Se5	71.168 (19)
Se4^i^—Nb1—Se1—Se2^ii^	117.956 (18)	Se2—Nb1—Se4—Se5	−37.53 (3)
Se2—Nb1—Se1—Se2^ii^	−153.63 (2)	Se5^i^—Nb1—Se4—Se5	119.734 (16)
Se5—Nb1—Se1—Se2^ii^	−155.98 (4)	Se1—Nb1—Se4—Se5	−149.25 (5)
Se4—Nb1—Se1—Se2^ii^	−25.33 (5)	Se3—Nb1—Se4—Se5	−79.423 (19)
Se5^i^—Nb1—Se1—Se2^ii^	65.416 (18)	Se3^ii^—Nb1—Se4—Se5	−156.37 (2)
Se3—Nb1—Se1—Se2^ii^	−99.124 (18)	V1^i^—Nb1—Se4—Se5	71.168 (19)
Se3^ii^—Nb1—Se1—Se2^ii^	−18.189 (16)	Nb1^i^—Nb1—Se4—Se5	71.168 (19)
V1^i^—Nb1—Se1—Se2^ii^	95.90 (4)	Se4^i^—Nb1—Se4—V1^i^	0.0
Nb1^i^—Nb1—Se1—Se2^ii^	95.90 (4)	Se2—Nb1—Se4—V1^i^	−108.70 (3)
Se4^i^—Nb1—Se1—Se2	−88.417 (19)	Se5—Nb1—Se4—V1^i^	−71.168 (19)
Se5—Nb1—Se1—Se2	−2.35 (4)	Se5^i^—Nb1—Se4—V1^i^	48.565 (15)
Se4—Nb1—Se1—Se2	128.30 (5)	Se1—Nb1—Se4—V1^i^	139.58 (5)
Se5^i^—Nb1—Se1—Se2	−140.96 (2)	Se3—Nb1—Se4—V1^i^	−150.59 (2)
Se3—Nb1—Se1—Se2	54.503 (16)	Se3^ii^—Nb1—Se4—V1^i^	132.46 (2)
Se3^ii^—Nb1—Se1—Se2	135.44 (2)	Nb1^i^—Nb1—Se4—V1^i^	0.0
V1^i^—Nb1—Se1—Se2	−110.47 (4)	Se4^i^—Nb1—Se4—Nb1^i^	0.0
Nb1^i^—Nb1—Se1—Se2	−110.47 (4)	Se2—Nb1—Se4—Nb1^i^	−108.70 (3)
Se4^i^—Nb1—Se2—Se3	−175.74 (2)	Se5—Nb1—Se4—Nb1^i^	−71.168 (19)
Se5—Nb1—Se2—Se3	−85.597 (19)	Se5^i^—Nb1—Se4—Nb1^i^	48.565 (15)
Se4—Nb1—Se2—Se3	−56.60 (3)	Se1—Nb1—Se4—Nb1^i^	139.58 (5)
Se5^i^—Nb1—Se2—Se3	155.39 (3)	Se3—Nb1—Se4—Nb1^i^	−150.59 (2)
Se1—Nb1—Se2—Se3	95.663 (19)	Se3^ii^—Nb1—Se4—Nb1^i^	132.46 (2)
Se3^ii^—Nb1—Se2—Se3	43.91 (2)	V1^i^—Nb1—Se4—Nb1^i^	0.0
V1^i^—Nb1—Se2—Se3	−130.41 (3)	V1^i^—Se4—Se5—Nb1	76.099 (17)
Nb1^i^—Nb1—Se2—Se3	−130.41 (3)	Nb1^i^—Se4—Se5—Nb1	76.099 (17)
Se4^i^—Nb1—Se2—Se1	88.598 (19)	Nb1^i^—Se4—Se5—V1^i^	0.0
Se5—Nb1—Se2—Se1	178.74 (2)	Nb1—Se4—Se5—V1^i^	−76.099 (17)
Se4—Nb1—Se2—Se1	−152.27 (3)	V1^i^—Se4—Se5—Nb1^i^	0.0
Se5^i^—Nb1—Se2—Se1	59.73 (3)	Nb1—Se4—Se5—Nb1^i^	−76.099 (17)
Se3—Nb1—Se2—Se1	−95.663 (19)	Se4^i^—Nb1—Se5—Se4	−119.319 (18)
Se3^ii^—Nb1—Se2—Se1	−51.75 (2)	Se2—Nb1—Se5—Se4	153.28 (2)
V1^i^—Nb1—Se2—Se1	133.92 (3)	Se5^i^—Nb1—Se5—Se4	−70.55 (2)
Nb1^i^—Nb1—Se2—Se1	133.92 (3)	Se1—Nb1—Se5—Se4	155.34 (4)
V1^ii^—Se1—Se2—Se3	20.533 (18)	Se3—Nb1—Se5—Se4	100.35 (2)
Nb1^ii^—Se1—Se2—Se3	20.533 (18)	Se3^ii^—Nb1—Se5—Se4	28.69 (2)
Nb1—Se1—Se2—Se3	−65.948 (18)	V1^i^—Nb1—Se5—Se4	−70.55 (2)
Se2^ii^—Se1—Se2—Se3	−18.564 (12)	Nb1^i^—Nb1—Se5—Se4	−70.55 (2)
V1^ii^—Se1—Se2—Nb1	86.48 (2)	Se4^i^—Nb1—Se5—V1^i^	−48.766 (16)
Nb1^ii^—Se1—Se2—Nb1	86.48 (2)	Se2—Nb1—Se5—V1^i^	−136.16 (2)
Se2^ii^—Se1—Se2—Nb1	47.384 (12)	Se4—Nb1—Se5—V1^i^	70.55 (2)
Se1—Se2—Se3—Nb1	60.808 (13)	Se5^i^—Nb1—Se5—V1^i^	0.0
Nb1—Se2—Se3—V1^ii^	−81.199 (17)	Se1—Nb1—Se5—V1^i^	−134.11 (5)
Se1—Se2—Se3—V1^ii^	−20.391 (16)	Se3—Nb1—Se5—V1^i^	170.90 (3)
Nb1—Se2—Se3—Nb1^ii^	−81.199 (17)	Se3^ii^—Nb1—Se5—V1^i^	99.24 (3)
Se1—Se2—Se3—Nb1^ii^	−20.391 (16)	Nb1^i^—Nb1—Se5—V1^i^	0.0
Se4^i^—Nb1—Se3—Se2	6.64 (3)	Se4^i^—Nb1—Se5—Nb1^i^	−48.766 (16)
Se5—Nb1—Se3—Se2	90.205 (19)	Se2—Nb1—Se5—Nb1^i^	−136.16 (2)
Se4—Nb1—Se3—Se2	141.87 (2)	Se4—Nb1—Se5—Nb1^i^	70.55 (2)
Se5^i^—Nb1—Se3—Se2	−115.45 (7)	Se5^i^—Nb1—Se5—Nb1^i^	0.0
Se1—Nb1—Se3—Se2	−63.036 (16)	Se1—Nb1—Se5—Nb1^i^	−134.11 (5)
Se3^ii^—Nb1—Se3—Se2	−141.981 (19)	Se3—Nb1—Se5—Nb1^i^	170.90 (3)
V1^i^—Nb1—Se3—Se2	102.13 (4)	Se3^ii^—Nb1—Se5—Nb1^i^	99.24 (3)
Nb1^i^—Nb1—Se3—Se2	102.13 (4)	V1^i^—Nb1—Se5—Nb1^i^	0.0
Se4^i^—Nb1—Se3—V1^ii^	109.33 (2)		

Symmetry codes: (i) −*x*+1/2, −*y*+1/2, −*z*+1; (ii) −*x*, *y*, −*z*+1/2.
